# Bridging the gap between omics research and dental practice

**DOI:** 10.1038/s41405-024-00199-3

**Published:** 2024-03-04

**Authors:** S. Kabbashi, IA. Roomaney, M. Chetty

**Affiliations:** Department of Craniofacial Biology, Pathology, and Radiology, Faculty of Dentistry, University of Western Cape, Cape Town, South Africa

**Keywords:** Oral diseases, Dentistry

## Abstract

**Aim:**

The burgeoning field of omics research has witnessed exponential growth in both medicine and dentistry. However, despite more than a decade of advancements, clinical dentistry, particularly in Low- and Middle-Income Countries (LMICs), has seen limited progress in integrating omics-based approaches into routine practice. This review aims to provide a comprehensive overview of the integration of omics approaches in dentistry, focusing on the challenges and opportunities for translating research findings into clinical practice.

**Methods:**

we conducted a literature review using key databases to provide a brief overview of the history of genomics in dentistry. Additionally, we summarised recent breakthroughs in omics relevant to oral health practitioners, emphasising the inadequate translation of omics research into clinical practice.

**Results:**

Despite significant growth in omics research in both medicine and dentistry, its translation into routine clinical practice in dentistry remains limited. We summarise recent breakthroughs in omics and highlight the gap between research advancements and clinical implementation.

**Discussion and conclusion:**

The integration of omics approaches holds promise for enhancing diagnostics, personalised treatment strategies, and preventive measures in dental practice, ushering in a new era of precision oral healthcare. However, several challenges, including infrastructure limitations, cost-effectiveness, and education gaps, hinder the widespread adoption of omics-based approaches in clinical dentistry. A strong commitment to transforming dentistry is required to embrace this transition. This shift has the potential to revolutionise oral healthcare by advancing precision diagnostics and treatment strategies tailored to individual patient needs.

## Introduction

Oral health is increasingly being recognised as an integral part of an individual’s overall systemic health; hence, oral health practitioners are required to respond to the ever-changing priorities and advancements in the scientific world. The dominant philosophy in dentistry is still traditional therapeutic dentistry, and the shift towards personalised clinical dentistry has yet to occur. The persistent and seemingly growing gap between research and translation in the field creates a disservice to researchers, dentists and, ultimately, the patients.

‘Omics’ is a term that has emerged to describe the field of large-scale data-rich biology and include areas of research such as proteomics, transcriptomics, genomics, metabolomics, lipidomics, and epigenomics. Omics is a rapidly growing, transdisciplinary domain that encompasses various fields such as genomics and epigenomics. It provides multi-level insights into biology that weren’t previously possible, allowing for an accelerated understanding of biological processes in both health and disease. However, understanding and implementing the learnings from omics requires a significant departure from conventional dental education and training. Despite the advocacy by various authors for integrating advanced biological sciences and components of omics into the dental curriculum [1–5], the adoption of these technologies has been sluggish to virtually non-existent in many dental schools [4, 6, 7]. It is predicted that by 2040, dental professionals will need to possess a very different and diverse set of skills compared to those required by dentists today [7]. Nevertheless, their training is not adequately preparing dentists to be relevant in this future world [7]. Many undergraduate dental programs barely touch the most basic genetic principles [4] despite growing evidence of the wealth of insights that omics provides into previously unanswerable questions in dentistry [8, 9].

Omics research can aid in understanding genes and proteins, their interactions, pathways, and networks responsible for the development and progression of oral diseases and disorders, ultimately opening doors to new therapies and preventative strategies. Dentists can no longer merely be clinicians, focussing only on the mouth [2] as our patients will demand a higher level of integrated care.

The evolution of omics research has raised pertinent questions about the integration of these advancements into dental care. A summary of the progress made in the fields of omics over the last decade is provided, and the imperative need for enhanced training in omics for all dental practitioners, is highlighted.

## The evolution of dental science

**Fig. 1:**
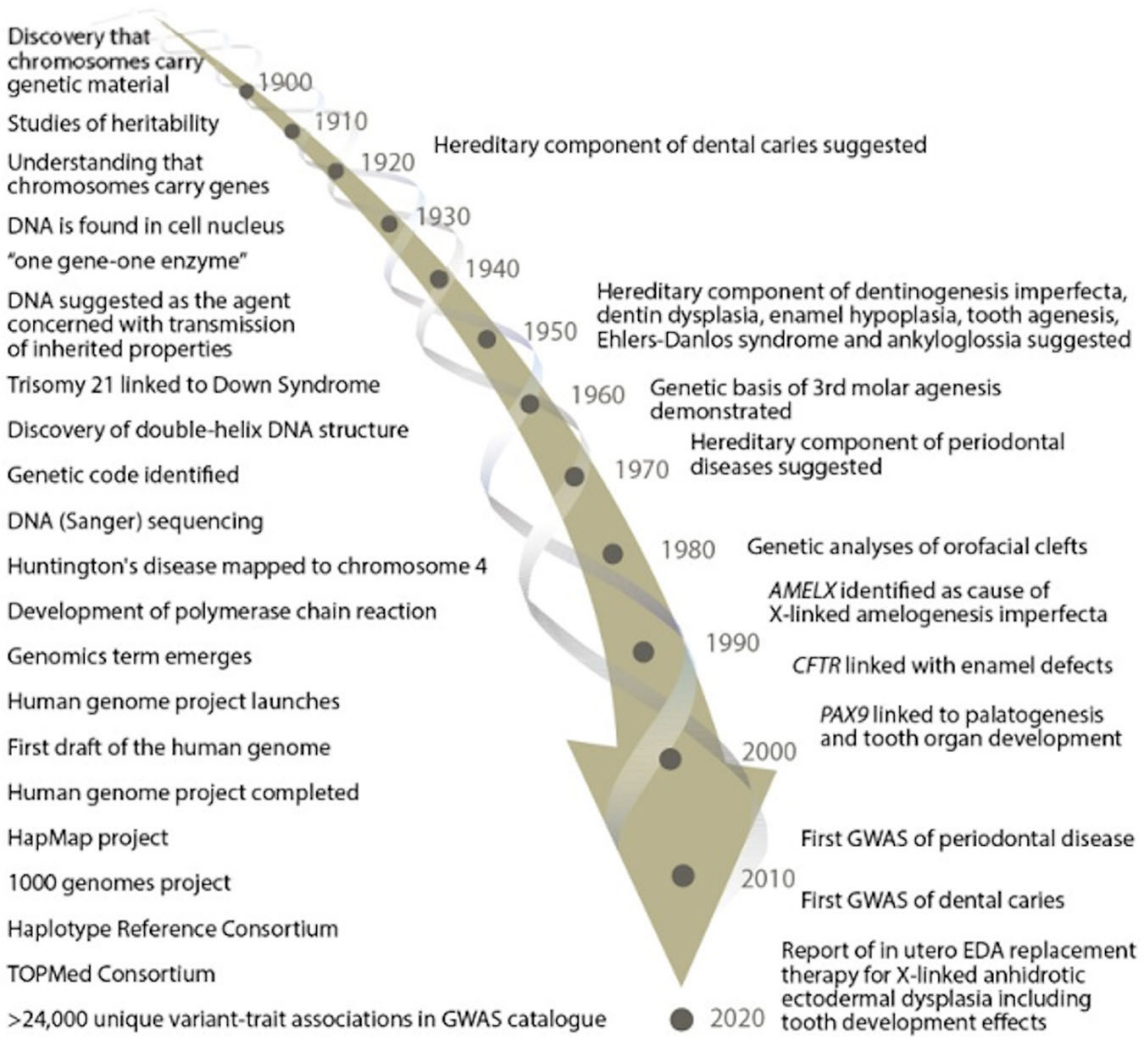
Timeline of genome research (left side) and oral health-specific landmark evolvements and illustrative reports (right side) since 1900). Reproduced from Divaris [11]. The Licensed Material is not part of the governing OA license.

The oral health domain has substantially benefited from the last century of advances in genetics and genomics. As early as the 1920s, authors started to observe the heritability of dental caries [10–13]. In 1956, an oral pathologist, Carl J. Witkop jnr, established the Human Genetics Section of the National Institute of Dental Research (NIDR) in the United States. He edited the book “Genetics and Dental Health”, exploring the genetic conditions with craniofacial and dental manifestations [14]. The 1950s also marked a breakthrough in molecular science with the discovery of the DNA double helix structure [15] published in 1953 and chromosomal abnormalities identified in 1959 [16]. Divaris provided a helpful timeline of oral health-related genomic research since the 1900s in Fig. 1 [11].

In the early 1990s, particular attention was given to the molecular genetic analysis of cariogenic microbes and the role of host genes in influencing susceptibility to caries [17]. Instead of the dominant one-size-fits-all approach to caries management and prevention, which was prevalent at the time, the concept of individual risk of dental decay began to develop. At the same time, there was significant advancements in cleft research with Karolyi and Erikson identifying a region on mouse Chromosome 3 homologous to human Chromosome 1q21, where variations were associated with significantly increased incidence of sporadic cleft lip or cleft lip and/or palate [18]. This information resulted in further investigations being carried out into the aetiology of cleft lip and/or palate.

Genetic and molecular experimental approaches such as cell lineage marking, monoclonal antibodies, organ culture and recombinant DNA techniques were fundamental to our understanding of the complex series of events governing craniofacial morphogenesis [19]. Although neural crest cells were discovered in 1868 [20], neural crest contributions to craniofacial embryology have only recently been accelerated by single-cell transcriptomics and epigenomics, which have confirmed theories of the neural crest gene regulatory network [21]. However, many questions remain regarding the migration, differentiation and specification of neural crest cells, which remain an active area of research. In addition, regulatory molecules and specific gene codes, such as homeobox-containing genes, which mediate the morphogenesis of the various craniofacial regions, have proved invaluable to our understanding of neurocristopathies and have allowed for the development of a new classification and the identification of previously unknown neurocristopathies [22].

Globally, there has been a recognition that dental research has expanded to include the entire craniofacial region. This paradigm shift is underscored by a redefined understanding of oral health, recognising its multifaceted nature and its intrinsic connection to general health and overall wellbeing. Consequently, dental organisations are realigning their priorities and resources in response to this expanded perspective. in 1998, the National Institute of Dental Research (NIDR) underwent a name change, rebranding itself as the National Institute for Dental and Craniofacial Research (NIDCR). This renaming accurately mirrored the institution’s widened research scope, indicative of the broader agenda it embraced. Further validating this trend, the World Dental Federation revised its definition of oral health in 2016 to encompass the multidimensional aspects of oral and craniofacial health. Notably, this revision highlighted the interconnectedness of oral health with broader health considerations [23]. As recently as June 2023, the International Association of Dental Research (IADR) expanded its name to the International Association for Dental, Oral, and Craniofacial Research. These organisational shifts signify a global transition towards a more comprehensive and holistic approach to oral healthcare reform.

As oral health advances further into the 21st century, dental science research is making remarkable progress. Notable strides in pathobiology, regenerative medicine, bio-engineering, and personalised medicine are reshaping the landscape of dentistry which has the potential of delivering improved dental care and heralding a distinct transformation in the field.

## Scientific progress in dental omics

Improved understanding in the fields of genetics and genomics have spurred the emergence of numerous omics disciplines and definitions of some of the omics is provided in Table 1. The information gained from integrating data from these technologies in specific research domains has led to the development of knowledge-based branches of omics, such as immunomics and microbiomics [24]. Consequently, the implementation of multi-omics in medical science guided the comprehensive understanding of the causal relationship between molecular signatures and the phenotypic manifestations of disorders and traits. This appreciation has aided in the elucidation of disease etiopathogenesis, enabling presymptomatic testing, and the development of more robust disease nosology, diagnosis, prevention, and the promise of effective treatments. A few recent breakthroughs in omics research pertinent to dental researchers and practitioners, is underscored.

**Table 1:** Overview of the various omics disciplines implicated in dentistry.

Genomics	A branch of molecular biology focuses on the structure, function, evolution, mapping, and editing of the entire genetic information of an organism. Various techniques, including standard methods like PCR-based analysis, as well as cutting-edge technologies such as genome-wide analyses, genotyping arrays, whole exome, and whole genome sequences (WES/WGS, are employed in this field [86].
Transcriptomics	The study of gene expression by investigating all aspects of RNA within a cell or organism, various forms of RNA, including rRNA and non-coding RNA such as micro-RNA and si-RNA, among others [87]. Recent advances have reshaped this field; however, two fundamental techniques, microarray and RNA sequencing (RNA-seq), are still commonly employed in transcriptomic analysis [88].
Proteomics	The study of the complete set of proteins within a cell, organ, or organism at a specific point in time [89]. Mass spectrometry is generally considered the gold standard technique for proteomics analysis and is widely used to analyse bodily fluids, including saliva [90].
Metagenomics	The study of microbial communities and their genomic material within natural living environments, including unculturable microorganisms, is conducted through functional gene screening or sequencing analysis [91]. Next-generation sequencing (NGS) technology is considered the gold standard in this field, employing two common strategies. The first targets specific genomic loci of microorganisms, such as 16S rRNA metagenomic sequencing. The second is shotgun sequencing, which sequences DNA from all types of microorganisms, including host DNA, simultaneously. Additionally, more advanced technology, third-generation sequencing, is utilised, producing substantially longer reads without requiring PCR amplification [92].
Metabolomics	A branch of molecular biology involves the measurement of low-molecular-weight metabolites in a biological sample. It provides insights into the dynamic responses of living organisms at the endpoints of causal pathways [93]. Saliva’s metabolic profile, in particular, has shown promise in identifying biomarkers for various oral diseases, notably cancer [94].
Epigenomics	The study investigates the complete set of epigenetic modifications in the genetic material of a cell [95]. This field primarily employs various sequencing techniques for assessing DNA methylation and utilises chromatin immunoprecipitation (ChIP) technologies to characterise key epigenetic mechanisms, including histone modification, chromatin compaction, and nuclear organisation. These approaches provide in-depth analysis and facilitate genome-wide visualisation of epigenome modifications [96].
Epitranscriptomics	An emerging field focuses on profiling epitranscriptomic RNA modifications, which are functionally relevant changes to the transcriptome. These changes do not involve alterations in the RNA sequence but have a significant impact on gene expression [24].
Epiproteomics	.Investigates the post-translational modifications occurring in both histone and non-histone proteins [24].
Intractomics	An interdisciplinary field bridging biology and bioinformatics, this discipline investigates the interactions among proteins and other cellular molecules, exploring the outcomes of these interactions most commonly DNA-RNA interactions [24].

Genomics is undoubtedly an immense field. Genetic variants causing genetic diseases are being identified at break-neck speed. As of January 2024, the number of phenotypes with known molecular bases is 7475, and the number of genes with phenotype-causing mutations is 4875 [25]. These include cancers and more than 700 genetic conditions with known craniofacial manifestations. Summarising advances in this complex field is challenging in a few sentences. These breakthroughs are leading to tangible improvements in clinical management, including cancer therapies and patient diagnostics.

The exploration of chronic diseases with polygenic underpinnings represents a field of research that is both inherently complex and relatively nascent in its development. Polygenic-risk scores (PRS), are quantifiable genetic risk scores based on the cumulative impact of genetic variants aimed at improving the prediction of risk to common chronic diseases [26]. While the number of genome-wide association studies (GWAS) regarding dental conditions is steadily growing, the clinical utility of these findings is still limited [27]. Nevertheless, individuals participating in such studies may soon be seeking dental professionals armed with genetic data, necessitating clinical interpretation.

GWAS plays a pivotal role in identifying and validating genetic variants and genes implicated in diverse dental conditions. This approach facilitates the exploration of broad genetic variations, transcending the confines of traditional candidate-gene studies. Applications span oral and craniofacial disorders such as periodontal disease, dental caries, cleft lip and palate, oral pain, temporomandibular dysfunction, and oral cancer [28]. Petty et al. conducted a GWAS investigating 932 individuals with deep carious lesions, delineating 24 loci exhibiting strong GWAS signals and identifying eight variants associated with an elevated risk of apical periodontitis [29]. Interestingly, the implicated genes are linked to immune regulation and bone metabolism, further highlighting the interplay of systemic factors in dental diseases. In this study, *PALLD*, a prominent gene associated with apical periodontitis, is also associated with heightened risks of pancreatic cancer and myocardial infarction. These discoveries of novel genomic contributions, coupled with the growing wealth of data, have laid the foundation for innovative methodological approaches aimed at unravelling causal relationships.

The use of biological samples such as saliva, dental biofilm, and gingival crevicular fluid (GCF), rather than only blood, has provided a wealth of data for the improved understanding of oral and craniofacial diseases and disorders [24]. The discovery of unique proteins in an individual’s saliva, among many advantages, made saliva practical to use as a biomarker reflecting inter-individual variability [30]. In recent years, there has been significant progress in the identification of salivary proteins through proteomics analysis. The number of identified proteins has increased from less than 20 to more than 1000 [31]. In 2018, Bostanci and colleagues succeeded in mapping the salivary proteome of a cohort with different periodontal statuses and discovered many less-known regulated human proteins by coupling mass spectrometry, label-free quantitative (LFQ)mass spectrometry proteomics, followed by selected-reaction monitoring (SRM)-targeted proteomic approaches. As a result, 119 clinically credible biomarkers were discovered, which have predictive value in distinguishing between periodontal health and disease. They also managed to identify ten differentially abundant salivary proteins that highlight the subtle differences between the state of periodontal health and gingivitis disease [30]. Furthermore, targeted proteomics successfully identified differential “proteotypes” represented by fifty proteins, which showed more than four-fold higher levels in individuals susceptible to developing gingivitis in a rapid response to dental plaque compared to slow responders [32–34].

Gene therapy and gene editing, through the development of CRISPR-associated protein (CRISPR-Cas) genome editing systems, is a rapidly expanding field of research and holds great potential for practical applications. CRISPR-Cas genome editing tools are among the most substantial advances in the life sciences in modern history. Single-dose gene therapies to correct pathogenic mutations have moved quickly from bench to bedside, with several therapeutics designed by CRISPR pioneers entering various stages of clinical investigation. This editing tool has achieved massive success due to its affordability and ease of use [35]. One pioneering oral-related study successfully utilised this technology to encode glucosyltransferases (Gtfs) protein, a key virulence factor of Streptococcus mutans that uses sucrose to synthesise extracellular matrix. By disrupting the production of the extracellular polysaccharide matrix involved in dental biofilm formation, this approach shows potential for the prevention of dental caries and periodontal diseases [28]. This technology was also employed to identify genes associated with oral cancer. A recent study emphasised the importance of the p75neurotrophin receptor (p75NTR) in the pathobiology of human tongue squamous carcinoma cells. The study used the CRISPR/Cas9 system to create p75NTR knockouts and successfully showed that this receptor could be a promising target for therapy against tongue cancer [36]. Genome-scale CRISPR-Cas9Knock-Out (GeCKO) libraries for oral squamous cell carcinoma (OSCC) cell lines showed that 43% of the cell lines were responsive to the combination therapy [37]. Despite the approval of checkpoint inhibitors, targeted therapies for OSCC are still limited. This underscores the importance of identifying new therapeutic targets and biomarkers to enable more precise patient selection for therapies [38].

The advent of these genetic technologies holds significant promise in revolutionising the landscape of maxillofacial surgery, particularly in addressing morbid conditions associated with syndromic craniosynostoses [39]. This subset of conditions, exemplified by Apert, Pfeiffer, Crouzon, and Muenke syndromes, are associated with mutations in fibroblast growth factor receptor genes. The recurrent nature of pathogenic mutations within these genes across affected families creates a distinctive opportunity for the development of “off-the-shelf” gene editing therapies, poised to correct these mutations in afflicted paediatric patients. The therapeutic potential of these interventions could reshape paediatric craniofacial surgery, potentially eliminating the need for midface advancement procedures in affected children. However, despite many animal studies [39], research of this nature has not as yet been translated into human clinical therapies.

Transcriptomics is progressing our understanding of pathobiological mechanisms driving development and disease. The transcriptome comprises all the RNA molecules produced by the genes in a particular cell or tissue at a specific time, providing a snapshot of the genes that are actively being expressed. Put simply, it offers insights into which genes are active and which are quiescent at any given time. Analyses, including microarrays and RNA sequencing (RNA-seq) are used to examine gene expression levels between different cells and tissues in a particular state. Micro-arrays are limited to pre-designed probes, whereas RNA-Seq provides averages of gene expression across cell masses rather than cell-specific information [40].

Transcriptomics is increasingly being used to understand diseases such as Sjorgen’s disease [41], Enamel Renal Syndrome [42], and even dental caries [43]. The overarching objective of most of these investigations is to pave the way toward potential therapeutic interventions. Transcriptomics in cancer research shows massive potential for clinical translation. Its uses include diagnostics [44], tracking disease progression, and gauging therapeutic responses [45, 46]. It is envisaged that in the move from exploration to implementation, these findings will likely form the foundation for standard care in the near future.

A similar trajectory is unfolding in the domain of regenerative dentistry, where scientists examine the intricacies of dental tissue-producing cells and the functioning of dental tissues themselves. Regenerative dentistry aims to replicate normal tissue development, fostering the prospect of tooth and tissue regeneration [40, 47, 48]. While numerous challenges still loom for the widespread realisation of whole tooth regeneration, the relentless research endeavours in this arena promise breakthroughs that will ultimately redefine dental healthcare.

One such breakthrough was the improvement of oligodontia in X-linked hypohidrotic ectodermal dysplasia cases by Schneider and colleagues [49]. This was achieved by protein replacement therapy with recombinant ectodysplasin in six cases prenatally and three shortly after birth. As a result, the three infants exhibited clinical improvements and demonstrated more-than-expected tooth buds [50]. They found that the impact of prenatal dosing on permanent teeth appears to be suboptimal and there was no corrective effects on the development of primary teeth which form during early embryogenesis. Their work is currently ongoing with refinements being made to dosing protocols.

Similarly, RNA therapeutics is a rapidly emerging field demonstrating an almost unlimited capacity to address clinical needs [51]. RNA therapeutics have the potential to act on targets that would conventionally be considered “undruggable”. They can be developed more quickly and cheaply than small molecules and recombinant proteins, and they can be rapidly adapted for personalisation or to target evolving pathogens [51]. In fact, scientists from Kyoto University are poised to start the first phase 1 human clinical trial assessing antibody and siRNA against *USAG-1* to treat anodontia [52, 53]. Uterine sensitisation–associated gene-1 (*USAG-1*) deficiency is associated with supernumery tooth formation via enhanced bone morphogenetic protein (BMP) signalling. Murashami-Suginami et al. demonstrated that alleviating congenital tooth agenesis stemming from diverse genetic anomalies in mice is achievable by impeding *USAG-1* activity, either through *USAG-1* knockout or the administration of anti-USAG-1 antibodies [53]. If successful, this may be one of the most promising dental breakthroughs in recent years.

Interpreting the complex and dynamic oral microbial community has gained new insight with the introduction of metagenomic technologies. The oral microbiome is a complex and ever-changing composition that varies based on factors such as the host’s immune response, oral hygiene habits, and overall systemic health. Whether targeted or shotgun, metagenomics has been employed in many people with dental infections, such as dental caries, periodontitis, apical periodontitis, and apical abscess. It was found that these microbial communities consist of highly diverse and mixed microbiota with differentially abundant taxa. The role of dysbiosis in periodontal disease has been well-established. In 2017, Ai et al. found a strong association between the loss of oral microbiota diversity and the progression of periodontal disease [54]. They also found three keystone species in addition to *P. gingivalis*, potentially mediating the progression of periodontitis. Our understanding of the role of the oral microbiome in other oral diseases is growing; however, even more fascinating, is the acknowledgement of the multidirectional link between the oral microbiome and systemic diseases.

Dysbiosis of the oral microbiome is associated with a spectrum of auto-immune diseases [55], systemic cancers [56, 57], adverse pregnancy outcomes [58], cardiovascular disease [59], neurodegenerative disease [60], and the list continues to grow. Metagenomic research is opening doors to both the possibility of targeted antimicrobial therapy and therapies for dysbiosis [61]. However, further longitudinal studies are needed and are being conducted [62, 63] to determine whether these shifts in the microbiome precede clinical signs of disease; this would allow the microbiome to be used to determine disease risk [61] and to determine if treating the dysbiosis will prevent systemic disease onset or progression.

It is acknowledged that genomics cannot provide complete answers, and while the interaction between the environment and genome is appreciated, it is not always fully understood. Epigenomics is considered the interface between the genome and the environment. It is the study of heritable changes in gene expression or cellular phenotypes caused by mechanisms other than the underlying DNA sequence [64]. These epigenetic mechanisms alter gene expression and include DNA methylation, posttranslational modification of histone proteins, and noncoding RNA (ncRNA) [65]. A decade ago epigenetics was declared as “a new frontier in dentistry” [66]; yet, our knowledge in the field is still in the fledgling phase. In a recent longitudinal study, the first to assess DNA methylation in childhood dental caries and hypomineralisation; differential methylation was identified in several genes which were associated with dental caries at birth and hypomineralisation at six years [67]. The authors suggested that these findings may aid in risk stratification and the development of biomarkers for caries risk at birth. There has also been significant interest in epigenomics and periodontal disease [68–70] and oral cancers [71]. The intricate interplay among the genome, host defences, epigenetic modifications, and microbiota interactions is evidently complex, and our comprehension thereof remains at an early stage, with much yet to be explored. Large-scale multi-omics projects such as ENCODE will assist in unravelling these intricacies and pave the way for enhanced therapies, disease prevention and management [70].

## Discussion

Oral, dental and craniofacial health is an essential component of health and well-being throughout the life span, but the evolution of health professions education and health care delivery systems have separated professional oral health care from overall health care. This separation has contributed to unmet dental needs and misdirected health care use. Fortunately, investments in science have led to discoveries that enhanced our knowledge and interventions to prevent and better manage oral and dental diseases. However, there is a gap between what is known and what is applied to personal, population and professional health care practices.

Science and technological advances have converged to create unprecedented opportunities for new discoveries, cures and therapies. However, it is important to consider whether the dental fraternity is indeed well-positioned to leverage from the science and technology resources available today. Although the profession has a remarkable history of innovative discovery; we continue to grapple with ways to prevent, diagnose, treat, and halt craniofacial disorders, dental caries, periodontal disease, oral cancer and orofacial pain. Oral diseases and disorders are now recognised as the most prevalent chronic conditions affecting human populations globally [72]. The burden of oral diseases primarily stems from complex conditions involving the interaction of multiple genes with environmental factors [73]. With the ever-evolving scientific knowledge and technologies, the greatest challenge that the dental fraternity currently faces is implementing and enhancing omics literacy, promoting individual and population oral health, and reducing disparities [74].

Although developed countries such as the United States, United Kingdom, Australia, Japan, and South Korea have already reported capacities in using OMIC technologies for disease prevention, prediction, diagnosis, treatment, as well as family counselling, many nations seem not to be catching up with the current trend. The challenges in omics field encompass the complex nature of the investigated diseases [75], the multidisciplinary aspect of omics technologies lacking sufficient professional expertise, and the difficulty in standardising research methodology and ensuring reproducibility [76]. These challenges contribute to the hurdles faced in translating omics research into clinical practice. Burke and Korngieble underscored the absence of acceptable evidence thresholds for various uses of genomics information as a key factor in creating this gap [77]. The issue of adequate evidence is likely to become even more controversial as variable omics approaches are adopted. The substantial gap highlighted in the translation of omics research into clinical practice extends beyond dentistry, as emphasised by Zhang et al. who outlined similar challenges faced by genomic medicine [76]. One notable challenge involves the imperative need to rapidly identify, assess, and prioritise “actionable” diagnostic and therapeutic approaches to keep pace with the advances in research [76]. The lack of sufficient genetics knowledge among point of care physicians globally is another pressing issue [77].

Omics is a “big data” science that generates enormous amount sequence information, especially when the platform is NGS. Expertise, expensive and extensive computing facilities, broadband Internet connection, secured cloud computing, and stable power supply are required to store, access, manipulate, analyse, and interpret genomic data. These are not readily available in many countries.

Certainly, omics research has made remarkable strides within dental science over the past decade, and its rapid evolution promises to continue revolutionising healthcare. Despite these advancements, the prevalence of dental diseases remains alarmingly high in many countries [72]. Rather than narrowing, the gap between the laboratory and the dental chair seems to be widening. This trend is influenced by a multitude of factors [77] not unique to omics in dentistry but potentially exacerbated by perceived drawbacks such as complexity, costliness, a deviation from traditional dentistry taught in dental schools, and at present, limited benefit to patient care. Effectively bridging this divide and making omics research more accessible and applicable necessitates a comprehensive systemic shift involving stakeholders ranging from funders and dental researchers to policy-makers, practitioners, and patients. The responsibility lies in fostering a collective effort towards embracing change and ensuring that the benefits of omics research translate into tangible improvements in dental healthcare.

Dental clinician-scientists operating in Africa, for example, grapple daily with the daunting challenge of addressing this escalating oral health crisis amidst limited resources with the growing concern that current efforts are not effectively mitigating the crisis. Simultaneously, dental and medical research is progressing at such a rapid pace that, in developing countries, there is often a feeling that the research does not apply or is not applicable in their context. This dual challenge underscores the urgency of aligning cutting-edge research with the practicalities of daily work in addressing the pressing oral health needs of communities. While some say dentistry is not presently prepared for the idea of “personalised” dental public health, citing numerous health challenges and resource limitations, it is imperative to acknowledge that the concept of personalised dental public health, as defined, possesses the capacity to greatly augment the efficacy of public health initiatives. This is achieved through the customisation of these initiatives to align with the unique disease susceptibilities, risk factors, and environmental circumstances of diverse populations. Recognising the potential transformative impact of personalised dental public health, it becomes apparent that investing in this approach is not just a luxury but a critical necessity to address the oral health disparities and challenges. Dentistry ranks among the top professions for attracting the brightest and most talented of applicants, hence, moving towards the implementation of omics in dental care, the goal remains to improve patient outcomes and advance the field of oral health and general health. A few future imperatives and recommendations for change on how to enhance multi-omics research and translation, are provided.

1. Infrastructure and Resource Development: State-of-the-art research is costly and considerable improvement is required in the integration of dental research into general health research and healthcare provision. Working in silos is not appropriate or feasible as no part of the human body operates in isolation. The integration of dental research into general health research and healthcare provision is crucial for overall advancements in healthcare. There is a positive correlation between human capacity and the availability of research infrastructure. Investing in scientific equipment not only facilitates the training of students but also creates an enabling environment for scientists and researchers to conduct credible and high-quality research. Collaboration and breaking down silos are essential components for progress in research. Interdisciplinary cooperation can foster a more comprehensive understanding of complex issues, leading to innovative solutions.
  Using an example from South Africa, the joint agreement between South Africa and the European Union, lead to the development of the first edition of the South African Research Infrastructure Roadmap (SARIR) in 2016. This was a noteworthy initiative with the recognition that access to modern and relevant research infrastructure is vital for the quality and outcomes of research aligns with global best practices. In addition, the development of the SARIR was a crucial step toward fostering a competitive and sustainable national system of innovation. The strategic planning and framework outlined in SARIR for research infrastructure investment align with the long-term goals of enhancing research capabilities and contributing to global scientific collaborations. With the emphasis on financing options and participation in joint international research infrastructures reflects a proactive approach to ensuring South Africa’s integration into the global research landscape.
  The first, and most obvious reason, is that they allow funders to budget knowing what can be expected. Secondly, and most importantly, roadmaps force researchers to come together to share their ideas and work together for the national good. Thirdly, roadmaps make it possible for researchers to plan their own research in the reasonable knowledge of what will be supported. Roadmaps also inform institutions and other countries what is envisaged in the way of research initiatives and to plan their investments in human and support services accordingly.
  Given that biomedical science is highly globalised, it is necessary to establish and strengthen international science and technology networks and connections attracting skills where it is needed. Such endeavours are to be encouraged, given that highly specialised and large infrastructure cannot be duplicated at a regional or national level. Access to such infrastructure is therefore essential for researchers to be competitive.
2. Capacity Building and Education: The dental curriculum in many countries does not equip dentists for the future of dentistry [78]. To begin with, students at an undergraduate level must receive a solid foundation in biomedical science with exposure to topics such as genomics, proteomics, saliva diagnostics, bioinformatics and regenerative dentistry [1–3, 78]. In addition, formal training in clinical research methodology and exposure to meaningful scientific experiences are ways for students to acquire critical thinking skills and to create an early imprinting in the minds of BDS trainees about the need for studies that create the evidence to guide best practices of care for patients.
  The curriculum needs to be constantly adapted to include new knowledge. Promoting continuous education and training for dental practitioners and researchers is critical to enhancing their expertise in precision dentistry and multi-omics methodologies. “Teaching the teachers” must be prioritised to ensure that oral health educators are equipped with world-class knowledge and skills. Once example of this the free online course “Train the Trainer: Design Genomics and Bioinformatics Training” sponsored by Wellcome Connecting Science, which is one of several initiatives intended to upskill clinicians and scientists, including those in LMICs. These initiatives require investment and prioritisation from governments and universities. Workshops, seminars, and academic collaborations with well-established institutions can foster knowledge exchange and skills development in this rapidly evolving field. Dental schools must take a lead role in not only disseminating the latest information on emerging technologies to practicing clinicians but also as producers of new knowledge that can drive changes for dental practices.
3. Creating the dentist-scientist career pathway: A challenge, not unique but particularly prevalent in Africa, is that very few established programs currently empower dentists to pursue a career trajectory as dentist-scientists [79]. This void in education and professional opportunities highlights an urgent requisite: the need to cultivate comprehensive training pipelines and dedicated programs tailored to dentist-scientists’ specific needs and aspirations, particularly within the local contexts. Creating the dentist-scientist pathway aligns with the imperative of capacity building and holds the promise of retaining these professionals within Africa and other LMICs, avoiding their migration to more developed countries. This initiative is not merely an investment in individuals but a strategic move to bolster oral healthcare research, innovation, and practice.
  The profession has grown increasingly dependent on faculty members with PhDs who are committed to academic careers in dental schools. Unfortunately, the number of PhD faculty members within dental schools has dwindled due to high demands on teaching and clinical responsibilities and increasing difficulty in securing and retaining research funding. There must be greater efficiency in terms of knowledge productivity, throughput, graduation rates and participation, with particular emphasis on the growth in PhD graduates, as these are the dominant drivers of new knowledge production. Therefore, it is time for dental schools to renew investments to enrich the pool of faculty members, who are either generalists or specialists, in tangible ways by providing them the training and the resources to conduct patient-oriented research.
  The establishment of this pathway would result in the cultivation of the next generation of dental leaders and fortify the foundation of oral healthcare in Africa and other LMICs, ultimately contributing to improved health outcomes and sustainable development in these regions and globally. In the past few decades, innovative funding mechanisms for clinician scientists have been made available from funding bodies such as the National institute of Health, the NIDCR and the South African Medical Research Council (SAMRC). These awards provide individual and institutional stipends for formalised research training and career development and have greatly enriched the pipeline of dental-scientist scholars. However, best practices are yet to be adopted to ensure the retention of BDS/PhD graduates as research-centred faculty members who are continually supported to be successful within the dental school environment.
4. Data Sharing and Integration: Duplicate efforts will result in misappropriation of limited resources. Facilitating data sharing and integration across different universities and healthcare institutions can help build comprehensive databases. This collective data pool can then be used for large-scale multi-omics studies, providing valuable insights into dental health and disease patterns specific to regional contexts.
  There is a lack of studies on ethnically diverse genomes which makes it challenging to fully understand the human genome in terms of health and disease. This results in the incorrect classification of variant pathogenicity and significantly limits the potential of genetic research being used in clinical practice [72, 80, 81]. Ultimately, negatively effecting patient management and worsening health inequalities [72]. These challenges have spurred the interest in conducting genomic research in the global South, especially Africa. Examples of studies include the Human Heredity and Health in Africa (H3Africa) program launched in 2012 [72, 82], and more recently, the launch of the 3 Million African Genomes Project (3MAG) in 2021 [83]. The advantages of conducting genetic studies in African populations are well-established. African genomes exhibit exceptional diversity owing to factors such as their “genomic age,” historical migrations, admixture, and the influence of natural selection [84]. The oral health fraternity has not been particularly active in these initiatives and even less so with other omics studies involving oral diseases. Moving forward, enhanced participation by the dental fraternity would benefit all stakeholders.
5. Ethical Considerations: Ethical issues around data genomic sharing have been raised [85]. It is mandatory to ensure that data is not exploited with little to no population benefit being incurred. All nations should establish robust ethical frameworks and regulatory guidelines to safeguard patient privacy, informed consent, and responsible use of omics data, ensuring compliance with international standards.

## Conclusion

While omics research continues to advance rapidly, integrating translational omics into dental practice, presents an ongoing challenge. Evidently, within the dental community, some are still observing the precision medicine wave from a distance, while others are poised to ride it to new horizons. Further research in omics in dentistry is imperative, but, incorporating this field into the education of dental students and practitioners, is of critical importance.

By equipping all members of the dental profession with the knowledge and skills necessary to embrace the new frontier in dentistry, this, collectively will contribute to enhanced oral health for people globally. The path forward requires collaborative efforts on a universal scale, allowing dentistry to harness the potential of omics, thus, improving health for all and reducing health disparities.
